# Neuroinflammatory and Redox Responses in a Rat Model of NTG-Induced Migraine

**DOI:** 10.3390/ijms27010026

**Published:** 2025-12-19

**Authors:** Anastasia A. Kochneva, Aleksey N. Ikrin, Natalia O. Fokeeva, Olga V. Yakovleva, Ksenia S. Bogatova, Aleksey V. Yakovlev, Elena Yu. Radomskaya, Margarita A. Khlystova, Veronika A. Katrukha, Kristina V. Vasilyeva, Andrei M. Karhov, Maxim A. Solotenkov, Aleksandr A. Moshchenko, Vsevolod V. Belousov, Ilya V. Fedotov, Pavel E. Musienko, Guzel F. Sitdikova, Dmitry S. Bilan, Elena V. Gerasimova

**Affiliations:** 1 Department of Neurobiology, Scientific Center of Genetics and Life Sciences, Sirius University of Science and Technology, Federal Territory of Sirius, 354340 Sochi, Russia; kochneva.a.a@yandex.ru (A.A.K.); ikrin.an@talantiuspeh.ru (A.N.I.); musienko.pe@talantiuspeh.ru (P.E.M.); sitdikovaguzel@gmail.com (G.F.S.); 2 Department of Human and Animal Physiology, Institute of Fundamental Medicine and Biology, Kazan Federal University, 420012 Kazan, Russia; olga.jakovleva@kpfu.ru (O.V.Y.); ksesbogatova@kpfu.ru (K.S.B.); aleksey.yakovlev@kpfu.ru (A.V.Y.); 3 M.M. Shemyakin and Yu.A. Ovchinnikov Institute of Bioorganic Chemistry, Russian Academy of Sciences, 117997 Moscow, Russia; margaritakhlystova119@gmail.com (M.A.K.); katrukhav@gmail.com (V.A.K.); tina902014@gmail.com (K.V.V.); akarchoff@gmail.com (A.M.K.); belousov@fccps.ru (V.V.B.); d.s.bilan@gmail.com (D.S.B.); 4 Federal Center of Brain Research and Neurotechnologies, Federal Medical Biological Agency, 117513 Moscow, Russia; ximero@yandex.ru; 5 Life Improvement by Future Technologies Center, 121205 Moscow, Russiafedotovilyaeg@mail.ru (I.V.F.); 6 Physics Department, M.V. Lomonosov Moscow State University, 119992 Moscow, Russia; 7 Neurocampus Institute (Institute of Neuroscience and Neurotechnologies), Pirogov Russian National Research Medical University, 117997 Moscow, Russia

**Keywords:** nitroglycerine, migraine, neuroinflammation, hydrogen peroxide, HyPer7, pH, SypHer3s, fiber-optic interface technology

## Abstract

Neuroinflammation is a common pathophysiological feature of many disorders affecting the central nervous system, including migraine—one of the most prevalent neurological conditions, which significantly impairs quality of life, particularly when it progresses to the chronic form. The aim of the present study was to analyze oxidative changes following a single administration of nitroglycerin (NTG), as well as to investigate alterations in the glial microenvironment and inflammatory processes induced by chronic NTG administration. Registration of biosensor signals (HyPer7 and SypHer3s) in vivo did not reveal changes in hydrogen peroxide levels or pH following single NTG administration in striatum and cortex. In contrast, analysis of chronic NTG administration indicates neuroinflammatory processes occurring in the thalamus and the dentate gyrus of the hippocampus, but not in the somatosensory cortex without disruption of the BBB and decreased degranulation of meningeal mast cells. We observed a decrease in the mRNA expression in the thalamic tissue of the neuroprotective transforming growth factor beta 1 gene and an increase in the expression of the pro-inflammatory interferon gamma. The regional specificity of neuroinflammation supports the suggestion that maladaptive changes in these structures could play a critical role in the transition from episodic to chronic migraine.

## 1. Introduction

About one in seven people worldwide suffer from the neurological disorder known as migraine [1]. Migraine attacks are characterized by headache lasting from 4 to 72 h [2], and are accompanied by a wide range of additional symptoms, including photophobia, scotomas, yawning, nausea, allodynia, and others [3,4,5]. The trigeminal system is widely recognized as a key structure involved in the pathophysiology of migraine, with its peripheral fibers innervating the meningeal and cerebral arteries of the dura mater [6,7]. Activation of the trigeminovascular system, particularly nociceptive pathways, leads to the release of pain-inducing and inflammatory mediators in the dura mater, including substance P, calcitonin gene-related peptide (CGRP), pituitary adenylate cyclase-activating polypeptide, and neurokinin A [6]. The release of vasoactive peptides leads to vasodilation and plasma extravasation in the dura mater [8]. Central proections of the trigeminovascular system includes trigeminal nuclei the thalamus, hypothalamus, and many areas of the cortex, including the somatosensory, primary visual, olfactory, and auditory cortex [9,10]. Activation of these areas is associated with migraine-related symptoms, including disturbances in sensory and motor functions as well as cognitive deficits [11].

Several animal models typically involved activation and sensitization of the trigeminal system are used to reproduce the complex neurovascular nature of migraine [12]. One of the simple and effective models of episodic and chronic migraine is based on the injections of nitroglycerin (NTG) [13]. NTG after administration is rapidly converted to nitric oxide (NO) in mitochondria, triggering a cascade that leads to vasodilation and a reduction in arterial blood pressure [14]. Intravenous NTG administration in human induces headache in migraine patients and cutaneous hyperalgesia due to sensitization of the peripheral trigeminal afferents and the activation of central trigemino-cervical neurons [15,16]. Hypersensitivity to NO underscores its pivotal role in headache generation, thereby validating the NTG-based animal model of migraine. NTG administration in animals induces behavioral changes, including mechanical allodynia, photophobia, reduced locomotor activity, and impaired cognitive performance [17,18]. It is believed that many of the listed effects of NO are associated with its ability to increase electrical activity of the trigeminal nerve [19]. Several clinical and animal studies provided direct or indirect evidence of increased blood–brain barrier (BBB) permeability in migraine [20,21,22,23,24]; however, BBB dysfunction in migraine is still a subject of debate. 

Activation of mast cells and glial cells in migraine may lead to neuroinflammation. By neuroinflammation, we mean an inflammatory response in the central nervous system mediated by inflammatory molecules such as cytokines, chemokines, and other compounds. Neuroinflammation is a natural response to infectious and non-infectious stimuli; however, its protective function can shift into a maladaptive pathological state. Thus, chronic pain can be maintained by persistent neuroinflammation [25]. For this reason, the contribution of neuroinflammation to migraine pathophysiology remains an open question. Clinical studies on chemokines and cytokines have yielded mixed results: during migraine attacks, levels of pro-inflammatory mediators such as interleukin-1β (IL-1β), IL-6, and IL-8 increase, while levels of anti-inflammatory mediators both increase (IL-10) and decrease (IL-4 and IL-5) [26]. During attacks, patients also had elevated NO concentrations [27]. This inflammatory response is associated with both the peripheral immune system and glial cell activation.

Mast cells (MCs) of the dura mater are components of the immune system which respond to various triggers through activation and subsequent degranulation [28]. MCs contain a wide array of mediators, including histamine, serotonin, ATP, cytokines, substance P, prostaglandins, vasoactive intestinal peptide, and others [29]. Mast cells and trigeminal nociceptive terminals exhibit close anatomical and functional interactions which provide pathological feedback loop: MC degranulation induces activation of trigeminal afferents, which in turn release mediators that further stimulate mast cells [30,31]. This bidirectional crosstalk suggests that mast cell activation may contribute additional pro-inflammatory effects and disrupt vascular regulation in migraine, thereby playing a significant role in its pathophysiology.

Pathological processes driven by heightened neuronal and immune system activation during migraine attacks are also reflected in alterations of the glial microenvironment. Glial cells of the central nervous system comprise macroglial subtypes—including astrocytes, oligodendrocytes, and NG2-glia, alongside microglial cells, which represent the brain’s resident immune population [32,33]. An increased expression of inflammatory markers in microglia of the trigemino-cervical complex was shown in mouse models of chronic migraine [34] and a critical role for microglia have been proposed in the chronification of migraine [35]. Astrocytes may contribute to various phases of migraine attacks [36]. Thus, astrocytic calcium waves are directly involved in vasoconstriction following the cortical spreading depression (CSD)—correlate of migraine aura [37], and the initiation of aura in familial hemiplegic migraine type 2 may be triggered by astrocytic deficits in glutamate clearance [38]. Histological studies have demonstrated an increased glial fibrillary acidic protein (GFAP) staining of astrocytes in neocortical slices [39] and the entorhinal cortex [40] following CSD indicating astroglial activation. It is believed that pathological activation of glial cells contributes to the chronification of pain in various disorders, including migraine [35,41]. However, neuroinflammation does not occur uniformly throughout the brain. Existing studies on glial inflammation in migraine without aura have primarily focused on changes in the trigeminal nerve nuclei [42,43,44,45], in other brain regions involved in migraine, neuroinflammation has been scarcely investigated directly, with functional imaging methods predominating in the study of brain activity [46,47,48,49,50]. Despite the growing recognition of neuroinflammation in migraine pathogenesis, its contribution to the dysfunction of key cortical and subcortical structures remains insufficiently understood. A comprehensive analysis of neuroinflammatory changes in these regions may reveal novel pathophysiological mechanisms, which could be utilized in the diagnosis and treatment of migraine.

The aim of this study was to study neuroinflammatory processes in the rat brain, including the dynamics of oxidative stress, glial interactions, mast cell activation, and gene expression in a model of nitroglycerin-induced migraine.

## 2. Results

### 2.1. Mechanical Sensitivity Thresholds of Rats Following Acute and Chronic NTG Administration

**Figure 1:**
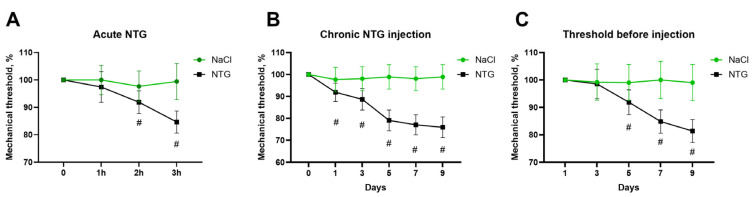
Effects of chronic nitroglycerine (NTG) administration on mechanical sensitivity thresholds. (**A**) Effect of a single acute administration of NTG and NaCl on the sensory thresholds of the plantar zone of the hind paws; (**B**) Baseline sensory thresholds measured before administration of NTG/NaCl. (**C**) Effects of chronic administration of NTG/NaCl, 2 h after injection. #—*p* < 0.05 compared to control measurement in group, Mann–Whitney *U* test.

To assess the development of hyperalgesia in the rat model of episodic and chronic migraine, mechanical nociceptive thresholds were measured before and after NTG administration using the von Frey test. An acute NTG administration is considered as a model of episodic migraine. Baseline mechanical sensitivity thresholds were measured before NTG/NaCl administration and again 2 h after each injection. The sensitivity threshold values before the first administration were taken as 100%. Chronic NTG administration led to a significant decrease in mechanical sensitivity thresholds 2 h after the first NTG injection, as well as after the 3rd, 4th, and 5th injections (*n* = 10, *p* < 0.05, Figure 1). Moreover, baseline sensitivity thresholds before NTG injection also showed a statistically significant decline on days 5, 7, and 9 of the experiment (*n* = 6, *p* < 0.05). In contrast, the control group receiving NaCl injections showed no changes in mechanical sensitivity (*p* > 0.05).

### 2.2. Effects of Acute Nitroglycerin Administration on H_2_O_2_ Level and pH Value in Rat Brain Neurons In Vivo

**Figure 2:**
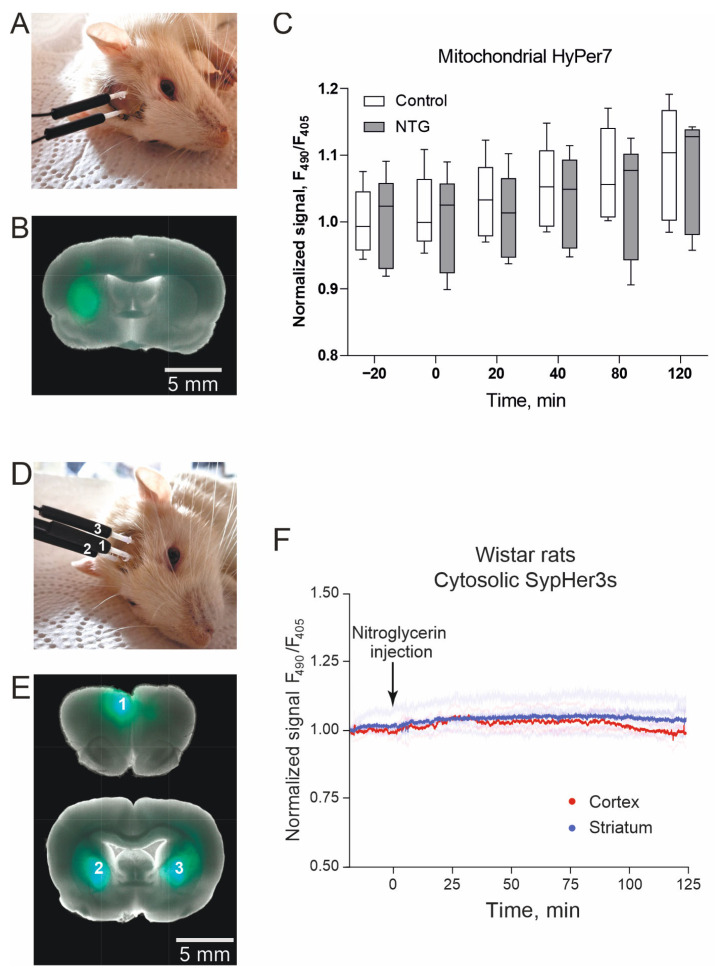
In vivo detection of biochemical parameters in rat brain tissues during the development of NTG-induced migraine. (**A**) Photograph of a rat with two implanted optical fibers in each hemisphere. Ceramic adapters containing the fibers are fixed to the skull using dental composite resin and acrylic cement. (**B**) Photograph of a rat brain slice with HyPer7 expression in striatum neurons. The image was obtained using a fluorescence microscope (F_490_). For clarity, an example of a brain slice is presented in which the injection of viral particles with the biosensor gene was carried out in the left hemisphere. No injection was made in the right hemisphere, which allows for the same fluorescence recording parameters to compare the target signal and tissue autofluorescence. (**C**) Dynamics of HyPer7 signal (F_490_/F_405_) in the mitochondrial matrix of striatum neurons in Wistar rats in the NTG-induced migraine model. Measurement values for animals injected with NTG (1 mg/mL, dose 10 mg/kg) are marked in grey (*n* = 3), and animals in the control group with saline administration are marked in white (*n* = 3). For (**C**) no significant changes were observed between groups at any time point. Two-way ANOVA. (**D**) Photograph of a rat with three implanted optical fibers (1—to the cortex, 2 and 3—to the striatum). (**E**) Photograph of a rat brain slice with SypHer3s expression in striatum and cortex neurons. The image was obtained using a fluorescence microscope (F_490_). (**F**) In vivo time-resolved registration of pH-biosensor SypHer3s signal (F_490_/F_405_) in neurons of the striatum and the cerebral cortex of Wistar rats in the NTG-induced migraine model. Pale colored lines mark the dynamics in each implanted fiber. Blue lines mark the dynamics of SypHer3s signal in striatum neurons (*n* = 6), red—in the cortex (*n* = 3). Lines averaged by groups are highlighted. The signal was recorded continuously during the entire experiment. Between the starting time point before the injection of NTG and 2 h later, no differences in the values of SypHer3s signal were observed. Paired *t*-test. Blue pale colored lines mark the dynamics of SypHer3s signal in striatum neurons (*n* = 6), red—in the cortex (*n* = 3). Lines averaged by groups are highlighted. Paired *t*-test.

To assess whether H_2_O_2_ is produced in neurons during migraine, we employed in vivo fiber-optic photometry to monitor the HyPer7 fluorescent signal in real time. HyPer7 is a genetically encoded biosensor that is sensitive to H_2_O_2_ [51]. Previously, we implemented this approach to record the dynamics of HyPer7 signal in vivo in rat brain tissues during the development of ischemic stroke in the acute phase of pathogenesis, as well as over several days [52,53,54,55]. Similar to our previous experience, the HyPer7 gene was delivered into Wistar rat brain tissues using adeno-associated viral (AAV) particles; we used serotype 9 (AAV9). To perform fluorescence signal recording in brain tissues of animals in vivo, we implanted optical fibers along the same brain coordinates where we injected the suspension of the virus. In this experiment, we recorded HyPer7 signal in striatum neurons (the caudate putamen structure). To localize the sensor, we used the common hSyn1 promoter [56]. We specifically monitored HyPer7 fluorescence dynamics in the mitochondrial matrix by localizing the sensor in this subcellular compartment using a signal peptide tag. The choice of mitochondrial HyPer7 targeting was determined by the observation in different models that H_2_O_2_ easily diffuses from the cytosol into the mitochondria, but not versa [51,57]. HyPer7 signal was detected in two symmetrical brain coordinates of the left and right brain hemispheres for each rat (Figure 2A). We confirmed the biosensor expression by its fluorescence in the brain tissue at the site of AAV injection at the end of the experiment for each animal (Figure 2B).

We recorded the fluorescent signal of HyPer7 biosensor in vivo in rat brain tissues using a custom optical system, the modification of which is presented in in [55]. HyPer7 is a ratiometric biosensor with fluorescence parameters: Ex._1_ 400 nm/Em. 516 nm and Ex._2_ 499 nm/Em. 516 nm [51]. Exciting light was supplied by diodes emitting at 405 and 490 nm through optical fibers implanted in the animal’s brain. The fluorescence emission was recorded in the opposite direction through the same fibers. Fluorescence passed through a bandpass filter (525/50) to a CCD camera. Thus, registration was performed via two fluorescent channels in each fiber, HyPer7 signal was calculated as the ratio of the fluorescence signal excited at 490 nm to the signal excited at 405 nm (F_490_/F_405_).

Before the experiment, the animals were anesthetized (urethane, 1.5 g/kg). We recorded the HyPer7 signal immediately after anesthesia of the animal and 20 min later. Having made sure that the sensor signal was stable, we stimulated migraine with an i.p. injection of nitroglycerin (concentration 1 mg/mL, dose 10 mg/kg). When the animal was at rest, HyPer7 signal was recorded in both hemispheres for 2 h with an interval of 20 and 40 min. At each time point, the F_490_/F_405_ ratio was recorded for 2 min with an interval of 2 s, based on which the average value was calculated. Throughout the entire measurement period after NTG administration, we did not detect any significant changes in HyPer7 signal. The signal values in the experimental group of animals did not differ from the control group, where saline solution was introduced (Figure 2C). 

We also investigated real-time pH dynamics in this model, since migraine is associated with the development of spreading depression [58]. The brain coordinates for injection and implantation of optical fibers chosen were the same (the area of the caudate putamen structure in each hemisphere). However, since depolarization affects the cortex, we established a third point for signal detection by implanting the third fiber into the cortex of the left hemisphere (Figure 2F). SypHer3s demonstrates the same optical characteristics as HyPer7, so the fluorescent signal (F490/F405) was recorded using the same settings of the optical system. Cell depolarization implies fast events, so SypHer3s signal was recorded continuously at 2 s intervals for 2 h from the moment of nitroglycerin injection (concentration 1 mg/mL, dose 10 mg/kg). We did not detect pH changes in neurons of the striatum and the cerebral cortex of urethane (1.5 g/kg) anesthetized Wistar in this model (Figure 2F).

### 2.3. Analysis of Mast Cell Activation After Chronic NTG Administration

Mast cell (MC) activation and subsequent degranulation are closely associated with sensitization and inflammatory processes in migraine [59]. The degranulation index (DI) reflects the extent of granule release containing pro-inflammatory molecules from meningeal mast cells in response to triggers such as activation of the trigeminal system, which can be observed in the dura mater.

**Figure 3:**
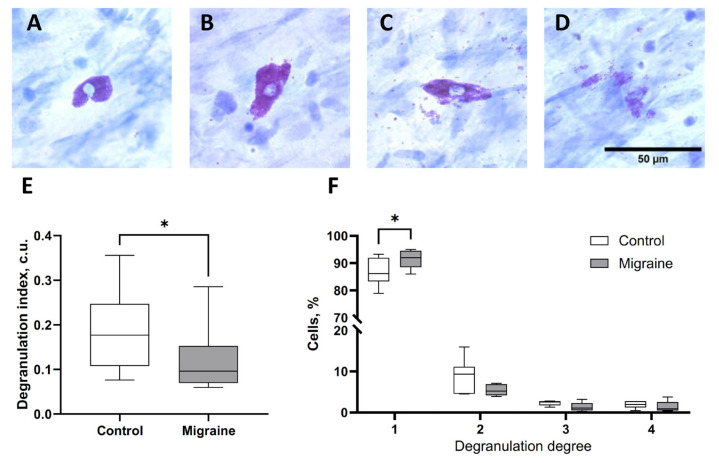
Assessment of mast cell activation in the dura mater based on the degranulation index. (**A**) Non-degranulated cells, (**B**) mild degree of degranulation, (**C**) moderate degree of degranulation, (**D**) high degree of degranulation, (**E**) comparison of the degranulation index between control and chronic migraine groups, (**F**) comparison of the proportion of mast cells at different degranulation degree. *—*p* < 0.05, Mann–Whitney *U* test.

In control animals, the degranulation index was 0.19 ± 0.08 a.u. (*n* = 7). In rats subjected to NTG-induced chronic migraine, the MC degranulation index was significantly lower, amounting to 0.12 ± 0.07 a.u. (*p* < 0.05, *n* = 5), which indicates its lower activity (Figure 3).

We also compared the proportion of MC at different stages of degranulation between the control group and the migraine model. The reduced degranulation index appears to be associated with a significantly higher proportion of non-degranulated mast cells in the NTG-treated group compared to controls (*p* < 0.05).

### 2.4. Glial Alterations in the Hippocampus Following Chronic NTG Administration

**Figure 4:**
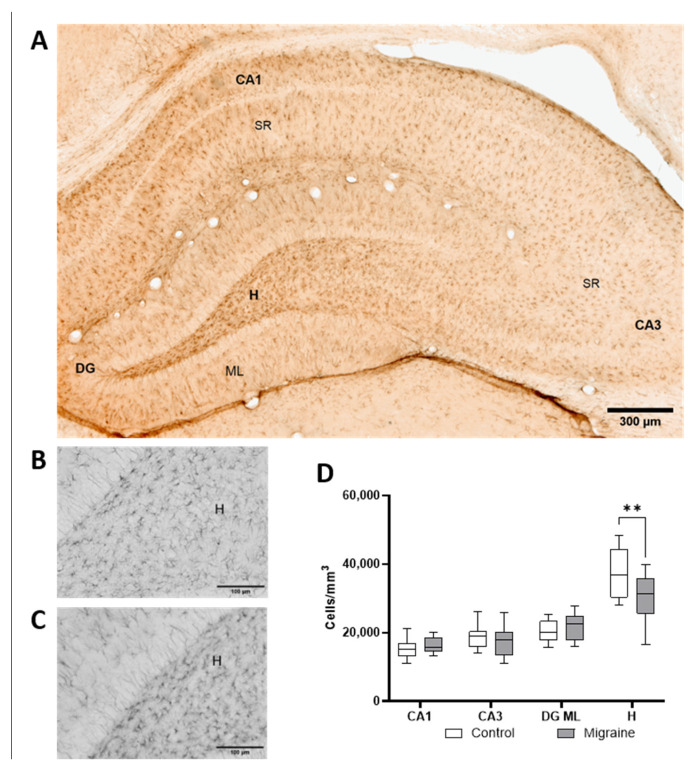
Analysis of GFAP immunostaining in astrocytes. (**A**) Microphotograph of the hippocampus in a control rat, with labeled regions: CA1 and CA3—hippocampal subfields, DG—dentate gyrus, SR—stratum radiatum, H—hilus, ML—molecular layer. Representative images of the hilus in control rats (**B**) and after NTG administration (**C**). (**D**) Quantitative comparison of cell density per unit area. **—*p* < 0.01, two-way ANOVA.

To assess changes in astrocyte population following the induction of chronic migraine in animals, immunohistochemical labeling with antibodies against GFAP and S100β was performed (Figure 4). We used GFAP as a general astrocyte marker to assess changes in cell morphology and distribution in response to chronic migraine from the perspective of cytoarchitectonics, while their functional implications were evaluated through blood–brain barrier permeability analysis described below. S100β was used as a marker of pro-inflammatory astrocyte phenotype, which is associated with various pathological conditions. In migraine, elevated levels of S100β in blood have been proposed as a diagnostic biomarker [60].

In control animals, the density of GFAP+ astrocytes (Figure 4D) in the CA1 region of the hippocampus was 15,417 ± 2809 cells/mm^3^, in the CA3 region—20,060 ± 5447 cells/mm^3^, in the DG ML (molecular layer of the dentate gyrus)—20,454 ± 3115 cells/mm^3^, and in the DG hilus—37,378 ± 6732 cells/mm^3^. Analysis of the stratum radiatum (SR) in the CA1 and CA3 regions and the molecular layer of the dentate gyrus (DG ML) revealed no significant differences in the density of GFAP+ astrocytes (*p* > 0.05) following chronic migraine induction. In the NTG-treated group, the astrocyte density was 1272 ± 2396 cells/mm^3^ in CA1, 17,431 ± 4471 cells/mm^3^ in CA3, and 21,849 ± 4175 cells/mm^3^ in DG ML. However, in the hilus, the density of GFAP+ cells significantly decreased to 30,320 ± 7082 cells/mm^3^ (*p* < 0.01).

**Figure 5:**
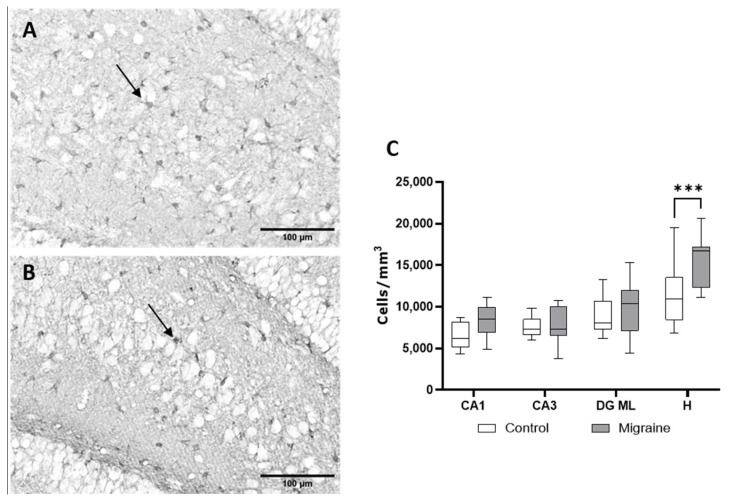
Analysis of S100β-positive astrocytes by immunohistochemical staining. (**A**) Hilus region in control rats, (**B**) hilus region after NTG administration; S100β-positive cells are indicated by arrows. (**C**) Quantitative comparison of cell density per unit area. ***—*p* < 0.01, two-way ANOVA.

Analysis of S100β+ astrocytes (Figure 5) also revealed a significant increase in cell density in the hilus (*p* < 0.001). In control animals, the density of pro-inflammatory astrocytes in the CA1 region was 6554 ± 1514 cells/mm^3^, in CA3—7488 ± 1149 cells/mm^3^, in the DG ML—8890 ± 2095 cells/mm^3^, and in the DG hilus—12,218 ± 3552 cells/mm^3^. Following chronic NTG administration, the density of S100β+ cells increased to 8370 ± 1980 cells/mm^3^ in CA1, 7785 ± 2231 cells/mm^3^ in CA3, 9918 ± 3341 cells/mm^3^ in DG ML, and 15,312 ± 3150 cells/mm^3^ in the DG hilus. Thus, while the number of activated pro-inflammatory astrocytes (S100β+) increased in the hilus, the overall density of mature astrocytes (GFAP+) in this region decreased after chronic NTG administration, suggesting a phenotypic shift in the astrocytic population toward a reactive, pro-inflammatory state rather than a global increase in astrocyte numbers. Enhanced inflammatory response in the hilus may contribute to memory impairments in rats with chronic migraine [18]. This region is involved in cell proliferation and plays a critical role in hippocampal cognitive functions.

**Figure 6:**
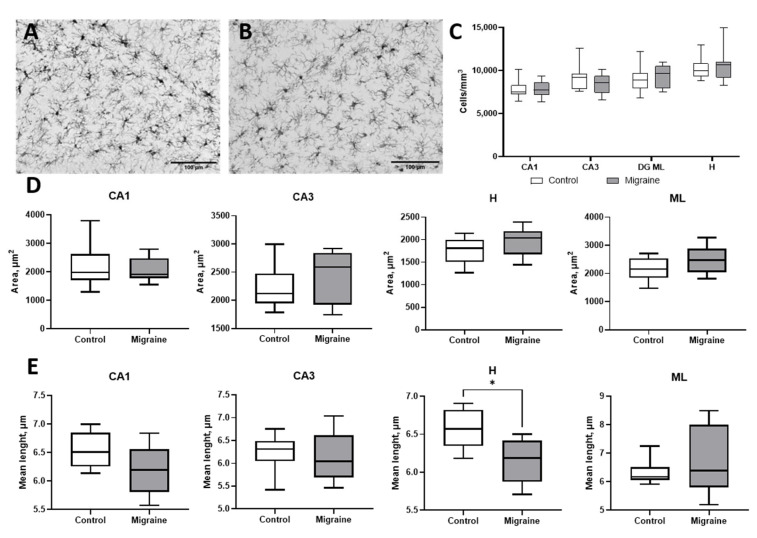
Analysis of Iba-1-positive microglial cells by immunohistochemical staining. (**A**) Hilus region in control rats; (**B**) hilus region in migraine model; (**C**) comparison of cell density per unit area; (**D**) average area of individual cells; (**E**) average length of cellular processes. *—*p* < 0.05.

Microglial cells are among the first responders in neuroinflammatory processes. We used Iba-1 to label microglial cells, as this common marker enables not only the assessment of cell density but also the analysis of their morphology. However, Iba-1 immunostaining (Figure 6) did not reveal any statistically significant changes in microglial cell density in any of the analyzed hippocampal layers (*p* > 0.05, two-way ANOVA). In control animals, the density of microglial cells was 7213 ± 2231 cells/mm^3^ in the CA1 region, 9128 ± 1417 cells/mm^3^ in CA3, 8461 ± 2826 cells/mm^3^ in the DG ML, and 9475 ± 2907 cells/mm^3^ in the hilus. Following chronic NTG administration, microglial cell density was 7814 ± 944 cells/mm^3^ in CA1, 8452 ± 1158 cells/mm^3^ in CA3, 9413 ± 1308 cells/mm^3^ in DG ML, and 11,494 ± 3229 cells/mm^3^ in the hilus. Although a numerical increase in microglial density was observed in the hilus, it did not reach statistical significance, suggesting that microglial activation may not be a dominant feature in the hippocampal neuroinflammatory response under the present experimental model of chronic migraine.

Morphometric analysis of microglia did not reveal changes in cell body area within the hippocampus (Figure 6D), but showed a significant reduction in the average length of cellular processes in the hilus region in the migraine model: 6.56 ± 0.25 μm in control and 6.15 ± 0.3 μm in migraine group (*p* < 0.05, Mann–Whitney *U* test). This finding may indicate a shift toward a pro-inflammatory microglial phenotype, as shortened processes are commonly associated with microglial activation and transition to a reactive state in response to neuroinflammatory stimuli. Analysis of microglia further confirms that the hilus is the most sensitive region to chronic migraine and undergoes glial remodeling.

### 2.5. Glial Responses in the Somatosensory Cortex of Rats with Chronic NTG Administration

**Figure 7:**
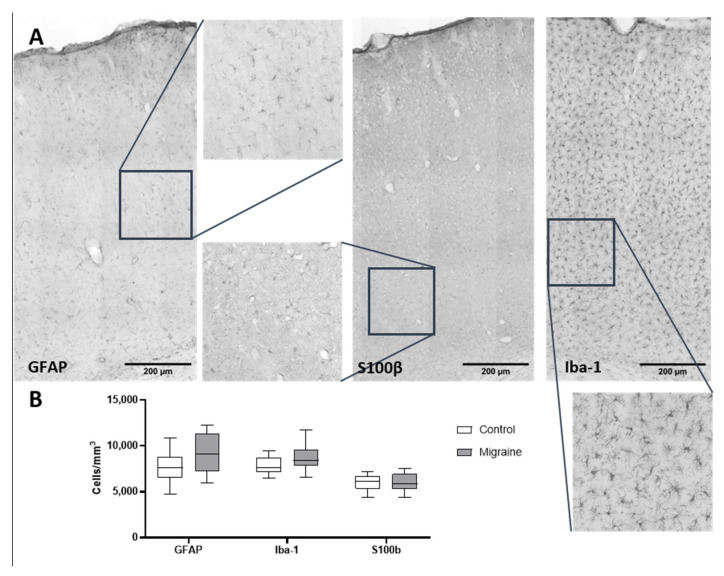
Analysis of cell density in the primary somatosensory cortex of Wistar rats. (**A**) Immunohistochemical staining of brain sections from control rats for GFAP, S100β, and Iba-1 markers. Dashed rectangles indicate the magnified areas shown in inserts. (**B**) Quantitative comparison of cell density per unit area.

Given that migraine is associated with alterations in somatosensory processing, we selected the primary somatosensory cortex (S1) as a region of interest to assess neuroinflammatory changes. We evaluated the density of astrocytes and microglial cells in this area (Figure 7). The density of GFAP-positive astrocytes in control rats was 7755 ± 1645 cells/mm^3^. Following chronic NTG administration, the density increased up to 9181 ± 2195 cells/mm^3^, but this difference was not statistically significant (*p* > 0.05).

Similarly, the density of pro-inflammatory S100β-positive astrocytes showed no significant change: 6029 ± 874 cells/mm^3^ in the control group versus 5992 ± 1045 cells/mm^3^ in the NTG-treated group (*p* > 0.05).

The density of microglial cells did not differ significantly between the control group and the NTG-treated group, amounting to 7861 ± 963 cells/mm^3^ and 8674 ± 496 cells/mm^3^, respectively (*p* > 0.05) (Figure 7). These findings suggest that alterations in somatosensory processing during migraine may not be primarily driven by local neuroinflammatory changes in the primary somatosensory cortex. Instead, they may be associated with functional or structural modifications in downstream components of sensory pathways, such as the thalamus or brainstem nuclei.

### 2.6. Glial Cells in the Ventral Thalamic Nuclei After Chronic NTG Administration

**Figure 8:**
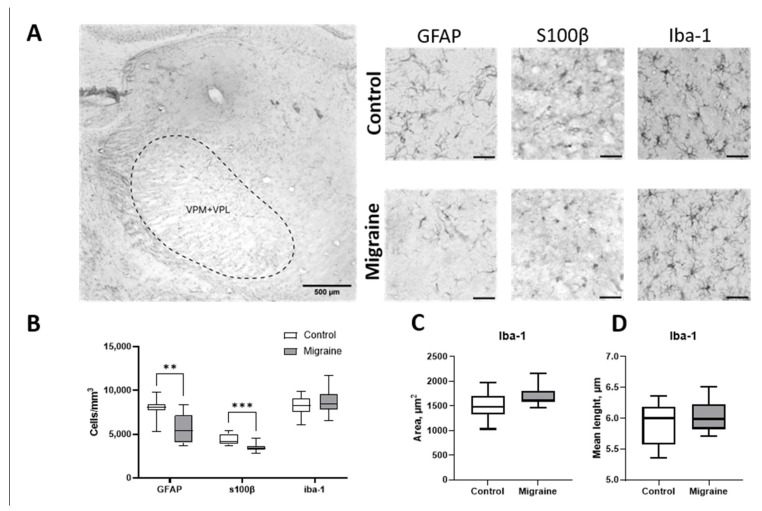
Analysis of astrocyte immunostaining in the ventral thalamic nuclei of Wistar rats in migraine model. (**A**) Glial cell staining in control and migraine groups; scale bar (right panels) = 40 μm. (**B**) Comparison of astrocyte and microglial cell density per unit area. (**C**) Average area of microglial cells. (**D**) Average process length. **—*p* < 0.01, ***—*p* < 0.001, Mann–Whitney *U* test.

Since our aim was to further investigate the ascending nociceptive pathway, we also investigated the ventral thalamic nuclei as a key structure, a relay station for somatosensory pain transmission. Analysis of the ventral thalamic nuclei following GFAP immunolabeling revealed a significant reduction in astrocyte density (Figure 8). In control Wistar rats, the density of GFAP-positive astrocytes was 8389 ± 1637 cells/mm^3^, whereas in animals with NTG-induced chronic migraine, the density decreased to 5665 ± 1654 cells/mm^3^ (*p* < 0.01).

Immunostaining for S100β+ astrocytes also showed a decrease in cell density following chronic NTG administration. In the control group, the density of pro-inflammatory astrocytes was 4408 ± 630 cells/mm^3^, while in the NTG-treated group, it was reduced to 3482 ± 492 cells/mm^3^ (*p* < 0.001).

Immunostaining for Iba-1-positive microglial cells in the ventral thalamic nuclei revealed no significant differences in cell density between groups (Figure 8B). In the control group, microglial density was 8285 ± 1028 cells/mm^3^, compared to 7635 ± 1207 cells/mm^3^ after chronic NTG administration (*n* = 5, *p* > 0.05). Analysis of microglial morphology also revealed no changes in the area or mean length of cell processes (Figure 8C,D). These results suggest that, despite astrocytic alterations in the ventral thalamus, microglia do not exhibit significant numerical or structural changes in this model of chronic migraine, pointing to a more selective glial response predominantly involving astrocytes.

### 2.7. Changes in Blood–Brain Barrier (BBB) Permeability in the NTG-Induced Migraine Rat Model

In the control group, no extravasation of Evans Blue (EB) dye beyond cerebral vessels was observed. The EB concentration in brain tissue homogenates of control animals was 0.06 ± 0.02 μg/mg tissue (Supplementary Figure S1). Repeated NTG administration over 9 days did not result in a statistically significant increase in BBB permeability in the experimental group. The EB concentration in brain homogenates of NTG-treated rats was 0.10 ± 0.03 μg/mg tissue (*p* > 0.05).

### 2.8. Altered Gene Expression in Rat Brain After Chronic NTG

**Figure 9:**
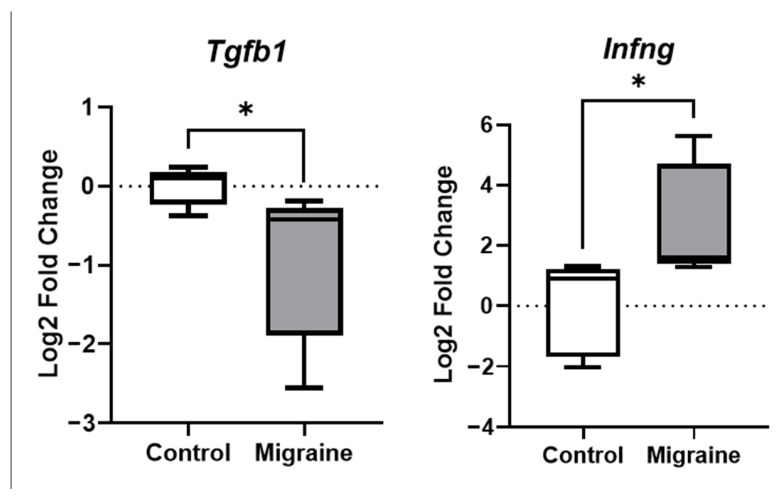
Altered mRNA expression of genes in the thalamus in chronic migraine. *Tgfb1*—Transforming growth factor beta 1, *Infng*—interferon-γ *—*p* < 0.05, Mann–Whitney *U* test.

Our analysis of gene expression in rats following repeated NTG injections revealed downregulation of *Tgfb1* mRNA (*p* < 0.05) and upregulation of *Ifng* mRNA (*p* < 0.05) in thalamic tissue (TH; Figure 9), compared to controls. No significant transcriptional changes occurred in the striatum (STR), hippocampus (HIP), primary somatosensory cortex (S1), or trigeminal nucleus caudalis (TNC) (Supplementary Figures S2–S6, Supplementary Table S1).

## 3. Discussion

In this study, we addressed the issue of oxidative stress and neuroinflammation in NTG-induced acute and chronic migraine. To this end, we used a well-established rodent model of chronic migraine based on NTG administration, which induces behavioral correlates of migraine, such as increased thermal sensitivity, development of cutaneous allodynia and hyperalgesia, photophobia, and migraine-like pain behavior, associated with activation and sensitization of neurons in the trigeminal complex [13,16,19,61,62,63]. In our study, the course of migraine modelling also showed a decrease in the thresholds of mechanical sensitivity, which leads to additional confirmation of the activation of the trigeminal system and the development of a migraine-like condition.

NTG leads to headache and migraine-like effects in patients, and is also widely used to induce a migraine-like condition in animals with acute and chronic administration [13,15]. Hydrogen peroxide (H_2_O_2_) is an important but not the only representative of reactive oxygen species (ROS). However, it is often H_2_O_2_ that serves as a marker of persistent oxidative stress in tissues, being the most stable compound among other ROS [64]. To determine whether H_2_O_2_ is generated in neurons in striatum during the development of migraine, which could indirectly indicate the development of oxidative stress, we used an in vivo approach of fiber-optic photometry of HyPer7 fluorescent signal in living rat brain tissues. Analysis of H_2_O_2_ dynamics did not reveal significant changes following NTG administration in Wistar. In addition, we analyzed the signal of the cytosolic biosensor SypHer3s in the cortex and striatum of Wistar, which is sensitive to pH changes, and found no alterations. Taken together, these data provide no evidence of oxidative stress or disruption of the mitochondrial redox balance. These findings further confirm the absence of CSD and the associated hypoxia that triggers it in the NTG-induced model of migraine [65,66].

Research in recent decades has shown the contribution and development of neuroinflammation in many diseases, from dementia [67] and Alzheimer’s disease [68] to stress and depression [69]. The term “neuroinflammation” is currently being criticized [70] due to the inaccuracy of the definition, but in a broad sense, neuroinflammation is understood as an inflammatory reaction in the central nervous system, characterized by the activation of both resident glial cells of the nervous tissue—astrocytes, microglia, oligodendrocytes—and infiltration of peripheral immune cells. Activation of the immune response leads to the release of inflammatory mediators—cytokines, chemokines, and an increase in the levels of reactive oxygen species [71]. Activation of microglia has also been shown in migraine, in particular, in the trigeminal ganglion, microglia were activated by the introduction of NTG, which led to the release of IL-1β [34,72]. Clinical studies suggest the possibility of using CX3C chemokine ligand 1, a marker of microglial activation, in the cerebrospinal fluid of patients as a potential biomarker of migraine [73].

Astrocytes play a direct role in neuroinflammation associated with migraine [36]. Astroglia perform multiple functions essential for maintaining neural tissue homeostasis, including the formation and modulation of the BBB [74]. GFAP is the most widely used marker for all astrocyte subpopulations; immunostaining for GFAP allows visualization of cell soma and the branching of major astrocytic processes [75]. Immunolabeling for the calcium-binding protein S100β specifically detects pro-inflammatory astrocytes in the central nervous system [76], although it primarily labels cell bodies and only partially stains cellular processes [75]. Clinical studies have reported elevated levels of S100β in the blood of patients with both episodic and chronic migraine [77,78]. A single administration of NTG to rats has also been shown to increase S100β levels [79], it was not used as a primary indicator of neuroinflammation in the brain regions selected for analysis in this study. We used GFAP and S100β together to differentiate changes in astrocyte activation (S100β+) from overall astrocyte density (GFAP+), providing a more specific readout of neuroinflammatory status. Although several markers, such as CD11β [80], CD68 and CD163 [81] and iNOS [82] have been used in migraine and neuroinflammation studies to identify functionally distinct microglial subsets, we selected Iba-1 as the sole microglial marker for this study. Iba1 reliably labels the entire microglial population (both resting and activated states) and, importantly, enables detailed morphological analysis that serve as sensitive proxies for functional activation.

In our study, we observed a reduction in the total number of mature astrocytes (GFAP+) alongside an increase in the population of pro-inflammatory astrocytes (S100β+) in the hilus region of the hippocampal dentate gyrus in rats after chronic NTG administration. These findings suggest NTG-induced astrocyte damage or phenotypic remodeling in the hippocampus following chronic NTG administration. The dentate gyrus is involved in information processing, receiving multimodal sensory input from the cortex [83], and is critically associated with cognitive functions [84]. The hilus contains proliferating neural progenitor cells—precursors of neurons, astrocytes, and oligodendrocytes—essential for the hippocampus-dependent functions such as learning and memory. This region is also considered a key modulator of excitatory signal transmission from the dentate gyrus to CA3 [85]. Disruption of glial homeostasis and neuroinflammation in this area may contribute to the cognitive deficits observed in migraine patients [86]. Despite these astrocytic alterations, no significant changes in BBB permeability were detected in our model. This finding aligns with clinical evidence, which similarly fails to support the hypothesis of BBB dysfunction during spontaneous or experimentally induced migraine attacks. However, because BBB permeability was assessed in whole-brain homogenates, the low regional resolution may have masked subtle, locally restricted alterations, particularly in areas with marked astrocytic dysfunction, such as the thalamus or hippocampus. Thus, neuroinflammatory changes in migraine may occur independently of structural BBB breakdown, highlighting the importance of glial activation as an early and potentially primary event in migraine pathophysiology [87].

In addition to alterations in astroglia, changes in microglial morphology were observed in the same hippocampal region-specifically, a significant shortening of average process length-without accompanying changes in cell body area or overall cell density. Previous studies on microglia in migraine have been limited and largely failed to detect cellular activation [88,89]. In our study, a more sensitive morphological analysis was applied, allowing for a detailed assessment of microglial changes that may not be apparent through cell density measurements alone. Morphological transformation is a well-recognized hallmark of microglial activation; however, studies report considerable heterogeneity in the morphological phenotypes associated with pro-inflammatory states. While process retraction is often interpreted as a sign of activation, it has also been linked to the M2-like (anti-inflammatory or repair-associated) phenotype in certain contexts [90]. Nonetheless, when considered alongside the observed astrocytic dysfunction—including reduced GFAP+ cell density and increased S100β+ reactive astrocytes—the morphological shift in microglia suggests the presence of a neuroinflammatory process in the dentate gyrus of the hippocampus in this model of chronic migraine. These combined glial changes suggest a complex, region-specific neuroinflammatory response that may contribute to hippocampal dysfunction and associated cognitive symptoms reported by migraine patients [91].

Neuroinflammation is recognized as an important component of migraine pathogenesis [92]. In rodent models of chronic migraine, repeated NTG injections induce headache, photophobia, and central sensitization of pain pathways, mimicking clinical manifestations of human migraine [12,93]. NTG functions as an NO donor, activating trigeminal nociceptive afferents and amplifying pain signals within the central nervous system [94]. Consequently, the trigeminothalamic pathway exhibits heightened neuronal activity in response to nociceptive stimulation. This pathway includes brainstem trigeminal nuclei, thalamic projecting neurons, and connections to the somatosensory cortex [10]. Notably, the thalamus serves as a critical hub for transmitting and modulating sensory pain signals [95]. In this study, no changes in glial cell distribution were observed in the primary somatosensory cortex, suggesting that chronic reorganization of pain circuits may occur at another level of the system. In the ventral thalamic nuclei, we found a reduction in the number of GFAP+ astrocytes. The simultaneous decrease in pro-inflammatory S100β+ astrocytes may be a consequence of a proportional reduction in the total astrocyte population. Microglial cells, however, showed no changes in morphology or tissue migration. Thus, impaired astrocytic function in the ventral thalamus may contribute to the development of tactile hypersensitivity following chronic NTG administration.

A connection between mast cell (MC) and microglial activation is thought to be mediated by IL-1β, which is present in the granules of both mast cells and microglia [96] and, upon degranulation, can activate microglia [97,98]. MC granules containing pro-inflammatory and vasoactive mediators [29] may contribute to sensitization, mechanical and thermal hyperalgesia [30,31], as direct activation of the trigeminal system can trigger mast cell degranulation [31]. In this study, chronic NTG administration led to a reduced degranulation index (DI) of MC and increased the proportion of non-activated MC in rats. Low MC degranulation refers to a reduced release of substances like histamine, cytokines, and other inflammatory mediators from MC. This process usually happens when MC are activated by specific triggers, but if degranulation is low, it means fewer mediators are released, potentially leading to a diminished immune response or altered inflammation. Studies on meningeal MC have demonstrated their activation and degranulation in response to trigeminal system stimulation and induction of a migraine-like state [30,31]. However, these studies primarily focus on the effects of acute exposure. The findings obtained in the chronic migraine model may indicate the engagement of compensatory mechanisms in response to repeated stimulation. For instance, mast cell activation in the skin has been shown to be suppressed under conditions of chronic inflammation [99]. It should be noted that we assessed the degree of degranulation not during or immediately after an induced migraine-like attack, but on the day following the last injection. Therefore, these data should be interpreted specifically as changes in mast cell activity in the interictal period of chronic migraine, reflecting a long-term adaptation or dysregulation of meningeal immune responses rather than acute activation.

Despite the systemic effects of NTG, the absence of changes in expression of pro-inflammatory genes (*Il1b*, *Il6*, *Nos2*, *Tgfb1*, *Ifng*, *Vim*, *Il12a*, *Ptgs2*, *P2rx7*, and *Stat3*) and the cellular activation marker (*Fos*) in the striatum (Supplementary Figure S2), hippocampus (Supplementary Figure S3), primary somatosensory cortex (Supplementary Figure S4), and TNCi (Supplementary Figure S5) indicates that these regions do not mount a robust classical neuroinflammatory response. This observation suggests either lower susceptibility to sustained glial activation or the presence of effective protective mechanisms that suppress inflammatory processes in these areas. It is worth pointing out the methodological aspect of the study, that gene expression analysis was performed after the final NTG injection during the interictal-like phase, rather than during the acute attack. This timing may account for the absence of the expected c-Fos upregulation, given its transient expression profile, and necessitates caution when interpreting negative results for early neuronal activation markers.

Simultaneously, thalamus (Supplementary Figure S6) demonstrates a distinct neuroinflammatory signature characterized by elevated interferon-γ (*Ifng*) levels and reduced transforming growth factor-β1 (*Tgfb1*) expression. As the central hub of the ascending trigeminothalamic pathway, this region exhibits transcriptomic alterations indicative of microglial polarization toward a pro-inflammatory M1 phenotype [100,101]. *Ifng* activates Stat3-dependent transcription of pro-inflammatory mediators, while *Tgfb1* normally maintains microglial M2 polarization and anti-inflammatory homeostasis [102,103]. Thus, chronic NTG exposure induces thalamic neuroinflammation in this rodent migraine model.

## 4. Materials and Methods

### 4.1. Animals and Design of the Study 

The experiments were conducted on Wistar rats (P90–120). Animals were housed in a controlled environment with a temperature of 21–24 °C, a 12 h light-dark cycle, and ad libitum access to food and water. All experimental procedures were carried out in accordance with the European Directive 2010/63/EU and approved by the local ethics committees of Kazan Federal University (protocol No. 33, dated 25 November 2021) and Sirius University of Science and Technology (protocol No. 2, dated 2 May 2023) and the Institute of Bioorganic Chemistry of the Russian Academy of Sciences (IBCh RAS) (protocol no. 354, 12 September 2022).

**Figure 10:**
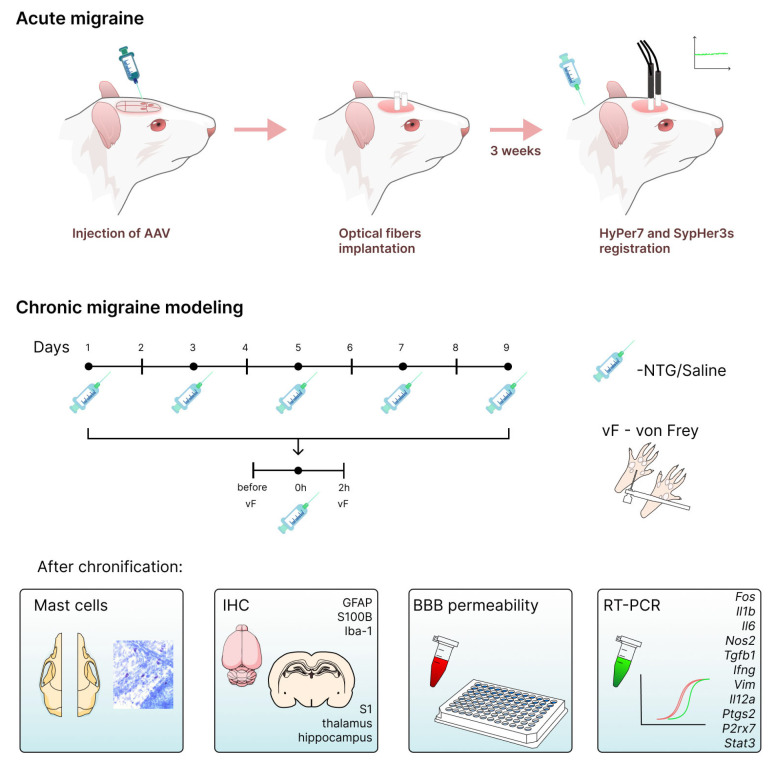
Schematic representation of the experimental design. The upper part illustrates the main steps in preparation for studying acute migraine using biosensors. Stereotaxic surgery and craniotomy were performed at selected coordinates for subsequent injection of AAV particles, illustrated here for SypHer3s. Optical fibers were implanted at the same coordinates, and ceramic ferrules were fixed using dental composite resin. Three weeks later, signals from HyPer7 and SypHer3s were recorded following NTG injection. The lower part of the figure presents a timeline depicting the schedule of NTG/saline injections and mechanical sensitivity testing using von Frey filaments. Tissue samples were collected after the final injection for subsequent molecular and histological analysis.

To induce a model of chronic migraine, male rats (*n* = 17) received intraperitoneal injections of nitroglycerin (NTG, Ozon, Zhigulevsk, Russia) at a concentration of 10 mg/kg in aqueous solution, according to the following protocol: five NTG injections administered every other day over a period of nine days (Figure 10). The control group (*n* = 25) received physiological saline in equivalent volumes. Mechanical sensitivity was recorded using von Frey filaments after the first, third and fifth injection. Meningeal mast staining, immunohistochemical study, BBB permeability and molecular analysis were performed after five NTG injections. H_2_O_2_ and pH were detected after one NTG injection.

### 4.2. Injection of AAV Particles and Implantation of Optical Fibers into the Rat Brain 

H_2_O_2_ generation was performed in rat brain in vivo using fiber-optic photometry of HyPer7 fluorescent signal. Genetic constructs hSyn1-HyPer7-MTS2 and hSyn1-SypHer3s-NES were delivered in rat brain using adeno-associated virus serotype 9 (AAV9). For both constructs the neuron specific promoter hSyn1 [56] was used. MTS2 tag (duplicated mitochondrial targeting sequence) [104] was used to localize HyPer7 biosensor to the mitochondrial matrix of neurons. NES tag (nucleus exclusion signal) [105] was used to localize SypHer3s in the cytosol. A titer of AAV9 with the constructs hSyn1-HyPer7-MTS2 and hSyn1-SypHer3s-NES was 1 × 10^13^ VG/mL. We used adult male and female Wistar rats—310 g. Surgical procedures were performed under 2% isoflurane (Miralek, Moskow, Russia) anesthesia using the SomnoSuite system (Kent Scientific, Torrington, CT, USA). We used the stereotaxic installation to fix the head, inject viral particles, and implant optical fibers. Prior to surgery, an injection of 0.25% bupivacaine (s.c.) was administered at the incision site, alongside an injection of ketoprofen (s.c.) for systemic analgesia. Two bilateral craniotomies were performed to deliver AAV9 carrying HyPer7 gene to the striatum. Three craniotomies were made for the AAV9-SypHer3s injections: two at the same coordinates as the HyPer7-targeted sites, and an additional one positioned more anteriorly. A 33-gauge needle coupled to a 5 µL Hamilton syringe was lowered 5.5 mm into the brain tissues (AP −0.9 mm, ML ±4.0 mm, DV −5.5 mm) and 1 µL of the AAV9 suspension was infused at a rate of 200 nL/min. For cortical injections, the needle was inserted to a depth of 2.1 mm (AP +3.2 mm, ML +1.0 mm, DV −2.1 mm) and the infusion rate was reduced to 100 nL/min. Immediately after injection, optical fibers (105/125, FG105UCA, Thorlabs Newton, NJ, USA) were implanted at the same coordinates. The ceramic ferrules were fixed with a light-cured dental composite resin (DentLight-Flow A3) and the ferrules, along with a reinforcing screw, were then cemented in place using acrylic dental cement. After the operation, the animals were placed in vivarium conditions under the supervision of veterinarians. We observed a pronounced fluorescent signal after 3 weeks.

### 4.3. Registration of HyPer7 and SypHer3s Signal in Rat Brain Tissue In Vivo After Nitroglycerin Injection Using a Fiber-Optic Neurointerface

For in vivo recording of the fluorescent signal of cpYFP-based HyPer7 and SypHer3s biosensors in deep brain structures of rats, we used the fiber-optic neurointerface technology. A detailed description of the circuit and characteristics of the optical setup is given in our previous works [52,53,54,55]. After the animals were anesthetized (0.8–1.0% isoflurane or 1.5 mg/kg urethane), optical cables (fibers inside the furcation tube) were connected to the implanted fibers through ceramic adapters. We used glass-clad multimode fibers with 105/125 core/cladding diameter (FG105UCA, Thorlabs, Newton, NJ, USA) both for implantation and as optical cables in the system. The excitation light sources were diodes with central wavelengths of 405 nm and 490 nm (M405F1b and M490F, Thorlabs Newton, NJ, USA). For better spectral isolation of two fluorescent channels F_405_ and F_490_, filters FESH450 (Thorlabs, Newton, NJ, USA) and ET480/20 (Chroma, Rockingham, VT, USA) were used, respectively. The average power of LEDs was kept below 1 µW. The fluorescent signal from the brain tissue with the expression of HyPer7 or SypHer3s entered the CCD camera (4070C-GE-TE, Thorlabs, Newton, NJ, USA), passing through a bandpass filter (525/50, Chroma, Rockingham, VT, USA). SypHer3s signal was recorded continuously for two hours at 2 s intervals. HyPer7 was recorded at 20 min intervals for a total of 140 min. At each time point, the signal was recorded for 2 min, then the average value for each point was calculated. The animals were injected with NTG i.p. 20 min after the start of recording.

### 4.4. Von Frey Test 

Mechanical sensitivity thresholds were assessed using von Frey filaments (Ugo Basile, Gemonio, Italy) according to the “up-down” method^37^, starting with a 0.4 g (3.61 g/mm^2^) filament as described earlier [19]. Animals were placed on an elevated platform with a mesh floor and allowed to acclimatize for 20–30 min before the start of the procedure. Measurements were performed on the two hind paws with 1–3 s interval Tactile plantar sensitivity was evaluated before NTG injections and at 1, 2, 3 h after the 1st injection and 2 h after 2, 3, 4 and 5 injections of NTG (Figure 10).

### 4.5. Assessment of Blood–Brain Barrier Permeability

To evaluate BBB permeability, albumin extravasation into brain tissue was assessed using Evans Blue (EB) dye in control (*n* = 13) and chronic NTG groups (*n* = 7). Evans Blue (10% solution in 0.9% saline, 2 mL/kg) was administered intravenously and allowed to circulate for 60 min. Detection of EB in brain homogenates was performed according to previously established protocols [106], using a Multiscan FS microplate reader (Thermo Scientific, Waltham, MA, USA). Quantification was based on calibration curves generated from a series of known EB concentrations ranging from 0.025 to 1000 mg/mL. All measurements were performed in triplicate or more, using animals from different litters to ensure reproducibility and minimize batch effects.

### 4.6. Histological Analysis. Tissue Preparation

Brain tissues were isolated after the end of the migraine modeling on the 10th day after behavioral testing (Figure 10). Rats were anesthetized with isoflurane (Iso-Nic, Chemical Iberica PV, Salamanca, Spain) and transcardially perfused using a peristaltic pump with 0.9% NaCl solution until liver clearing was achieved, followed by 4% paraformaldehyde (PFA) in phosphate buffer for tissue fixation until rigid. After decapitation, the skull bones were carefully cleaned, hemisected, and the brain was gently extracted and transferred into falcon tubes containing 4% PFA for post-fixation at +4 °C.

### 4.7. Meningeal Mast Cell Staining

Fixed skull samples were washed in phosphate-buffered saline (PBS, pH 7.4). The dura mater was carefully dissected from the skull halves, spread onto glass slides, and air-dried. Specimens were stained with 0.1% toluidine blue solution for 10 min, thoroughly rinsed with distilled water, dehydrated in 96% ethanol, and coverslipped. Slides were imaged using a Zeiss Axio Lab.A1 microscope equipped with a digital camera (Carl Zeiss, Thornwood, NY, USA). For each sample, at least 100 mast cells were analyzed across randomly selected fields of view. The degree of degranulation was assessed visually based on the number of extracellular granules surrounding mast cells. To quantify mast cell activation, the degranulation index (DI) was calculated according to the following formula [107]:

DI = (A × 0 + B × 1 +C × 2 + D × 3)/*n*, (1)

where A, B, C, and D are the degree of cell degranulation, where A—non-dergranulated mast cells, B—cells with a weak degree of degranulation (up to 5–7 granules near the mast cell, the boundaries are clearly visible), C—moderately degranulating mast cells (release of 7–20 granules), D—cells with a high degree of degranulation (more than 15 granules, not the boundaries of the mast cell); *n* = the number of all mast cells.

### 4.8. Immunohistochemical Staining of Glial Cells

Fixed rat brain samples were sectioned on a vibrating blade microtome (Leica VT1000S, Leica Biosystems, Vista, CA, USA) into 50 μm-thick coronal slices. Free-floating sections were collected and stored in cryoprotectant solution at –18 °C until use. Immunohistochemical staining was performed according to the protocol described by Stepanichev et al. [108]. To label mature astrocytes, primary rabbit polyclonal antibodies against GFAP were used at a dilution of 1:2000 (Abcam, Waltham, MA, USA). Activated astrocytes were detected with rabbit monoclonal antibodies against S100β at a dilution of 1:4000 (Abcam, Waltham, MA, USA). Microglial cells were visualized using rabbit polyclonal antibodies against Iba-1 (ionized calcium-binding adapter molecule 1) at a dilution of 1:500 (Abcam, Waltham, MA, USA). After incubation with primary antibodies, sections were washed in phosphate-buffered saline (PBS) containing 0.3% Triton X-100 (Himedia Laboratories, cat. MB031, Thane, Maharashtra, India). Subsequently, sections were processed using a secondary antibody kit with 3,3′-diaminobenzidine (DAB) as the chromogen (PrimeBioMed, cat. 78-310004-15, Moskow, Russia). Following DAB staining, sections were rinsed in distilled water, mounted onto glass slides, dehydrated in 98% ethanol, and coverslipped using VitroGel mounting medium (ErgoProduction, Saint Petersburg, Russia).

### 4.9. Histological Analysis

Sections of the dorsal hippocampus were imaged using a Zeiss Axio Lab.A1 microscope (Carl Zeiss, Thornwood, NY, USA) with ×20 objective. Identical regions were captured in both experimental groups: CA1 and CA3 *stratum radiatum* (SR), hilus, and the molecular layer of the *dentate gyrus* (DG ML) of the dorsal hippocampus, as well as the region of the ventral thalamic nuclei in control (*n* = 7) and chronic NTG group (*n* = 5). Primary somatosensory cortex (S1) sections were imaged using an automated imaging system, Celena X (Logos Biosystems, Anyang-si, South Korea), with a ×20 objective. For S1 analysis, a perpendicular line was drawn from the *corpus callosum* to the pial surface to define the region of interest, and cells were counted across all cortical layers along this axis.

Glial cell counting was performed using FIJI ImageJ V.1.54 (NIH, Bethesda, MD, USA). Only cells whose somata and processes were fully within the section thickness were included in the analysis. The density of GFAP+, S100β+, and Iba-1+ cells per mm^3^ was calculated using the following formula [109]:

d = (10^6^ × *n*)/(S × l), (2)

where *n*—the number of cells, S—the area of the analyzed region (in μm^2^), l—the section thickness (in μm), and 10^6^—the conversion factor from μm^2^ to mm^2^.

A freely available plugin [110] was also used for a more detailed analysis of microglia morphometry. We analyzed the average cell area (μm^2^) and average process length (μm).

### 4.10. Gene Expression

For RT-PCR gene expression analysis, we used 5 male rats subjected to chronic NTG administration (NTG group, *n* = 5) and 5 male control rats receiving NaCl (Control group, *n* = 5). Prior to decapitation, rats were euthanized with 3% isoflurane. Brains and trigeminal ganglia were rapidly dissected on ice, flash-frozen in liquid nitrogen, and stored at −80 °C until processing. Target brain regions (striatum, hippocampus, primary somatosensory cortex, thalamus and trigeminal nucleus caudalis) were microdissected in a cryostat at −20 °C to minimize mRNA degradation during tissue isolation.

Total RNA was extracted from frozen brain samples by manual homogenization in 1 mL of ExtractRNA reagent (Evrogen, Moskow, Russia), a monophasic solution of phenol and guanidine isothiocyanate. The lysate was incubated at room temperature for 10 min with intermittent vortexing. Following centrifugation at 12,500× *g* (4 °C) for 10 min, the supernatant was transferred to a new tube. For phase separation, 0.2 mL chloroform was added, and samples were vigorously shaken by hand for 15 sec. After centrifugation (12,500× *g*, 4 °C, 10 min), the aqueous phase was collected. RNA was precipitated by adding 0.5 mL isopropanol, mixing by pipetting, and centrifuging (12,500× *g*, 4 °C, 10 min). The RNA pellet was washed with 1 mL 80% ethanol, centrifuged (12,500× *g*, 4 °C, 5 min), and air-dried for 5–7 min. RNA was resuspended in nuclease-free water and incubated at 55–60 °C for 3–5 min to facilitate dissolution. RNA purity was assessed spectrophotometrically (A260/A280 and A260/A230 ratios) and by agarose gel electrophoresis. Genomic DNA contamination was removed using DNase E (Evrogen, Moskow, Russia). Purified RNA was stored at −20 °C. All procedures were performed on ice with minimal freeze–thaw cycles to prevent degradation.

First-strand complementary DNA (cDNA) synthesis was performed using the MMLV RT kit (Evrogen, Moskow, Russia) containing Moloney Murine Leukemia Virus reverse transcriptase (MMLV RT) expressed in E. coli. This system is designed for first-strand cDNA synthesis from single-stranded RNA templates, followed by applications in amplification, cloning, expression analysis, and quantitative real-time PCR (qPCR). Total RNA (2.5 μg) was combined with 2 μL oligo(dT) primers in nuclease-free water (total volume: 11 μL). Samples were denatured at 70 °C for 2 min to disrupt secondary structures and immediately cooled on ice. A 9 μL master mix containing 4 μL 5X First-Strand Buffer, 2 μL dNTP mix (10 mM each), 2 μL DTT (20 mM), and 1 μL MMLV RT (100 U/μL) was added to the cooled RNA-primer mixture. Reactions were mixed by pipetting, briefly centrifuged to eliminate air bubbles, and incubated at 37 °C for 60 min. Enzyme inactivation was achieved by heating at 70 °C for 10 min, followed by immediate cooling on ice. Synthesized first-strand cDNA was stored at −20 °C for subsequent qPCR analysis.

For this study, 11 target genes (*Fos*, *Il1b*, *Il6*, *Nos2*, *Tgfb1*, *Ifng*, *Vim*, *Il12a*, *Ptgs2*, *P2rx7 and Stat3*; see Table 1) were selected for quantitative real-time PCR (qPCR) analysis. *Actb* (β-actin) and *Gapdh* (glyceraldehyde-3-phosphate dehydrogenase) served as housekeeping genes [111]. Rat exon sequences of genes were retrieved from NCBI GenBank (https://www.ncbi.nlm.nih.gov/genbank/). Primer pairs spanning exon-exon junctions were designed using UGENE software V.53.0 to minimize dimer formation and stable secondary structures [112]. All primers were optimized for uniform melting temperatures (60 °C) and validated for specificity.

For real-time PCR, we used the ready-to-use 5X qPCRmix-HS SYBR reaction mix (Evrogen, Moskow, Russia) containing high-performance Taq DNA polymerase with monoclonal antibodies, SYBR Green I intercalating dye, dNTP mixture, Mg^2+^, and PCR buffer. The final 1X reaction mix contained 3 mM Mg^2+^ and 0.12 mM of each dNTP. The reaction mix was thawed at room temperature and thoroughly mixed. The 25 μL reaction volume contained 5× SYBR Green master mix (Evrogen, Moskow, Russia), 0.4 μM of each primer, and 50 ng of cDNA. DEPC-treated nuclease-free water was used as negative control instead of cDNA. Amplification was performed in triplicate using the Applied Biosystems QuantStudio 5 real-time PCR system (Thermo Fisher Scientific, Waltham, MA, USA) with the following cycling parameters: initial denaturation at 95 °C for 5 min; 40 cycles of denaturation (95 °C for 15 s), primer annealing (60 °C for 30 s), and extension (72 °C for 60 s); followed by a dissociation stage to generate a melt curve: 95 °C for 15 s, 60 °C for 60 s, and gradual heating to 95 °C at 0.05 °C/s with continuous fluorescence measurement.

### 4.11. Statistical Analysis

Statistical analysis was performed using Origin Pro (OriginLab Corp., Northampton, MA, USA), GraphPad Prism 10 (GraphPad Software, San Diego, CA, USA), and Microsoft Excel (Microsoft, Tulsa, OK, USA). Normality of distribution was assessed using the Shapiro–Wilk test, and homogeneity of variances was evaluated with the *F*-test. For comparisons between two independent groups, the Mann–Whitney *U* test was used. Two-way analysis of variance (ANOVA) was applied for analysis of multiple factors, followed by appropriate post hoc tests when necessary. For paired data, the Wilcoxon signed-rank test or paired *t*-test were employed as indicated in the ‘Results’ section and figure captions. Statistical significance was defined as *p* < 0.05. The value of (n) represents the number of animals per group. To compare more than three groups, we used the Kruskal–Wallis test. Data that shown as box-and-whisker plots: the box represents 25th-75th percentiles, the whiskers mark the minimum and maximum, the line inside the box—the median.

## 5. Conclusions

In this study, we demonstrated that chronic administration of NTG to rats is accompanied by a pronounced neuroinflammatory response, primarily localized in key subcortical structures-the ventral nuclei of the thalamus and the hilus of the dentate gyrus of the hippocampus. However, no changes in the redox balance (H_2_O_2_ and pH levels) or blood–brain barrier permeability were observed, indicating the absence of oxidative stress and structural disintegration of the central nervous system in this model. Meningeal mast cells showed a lower degree of degranulation in chronic migraine, which may be an adaptive response. These data indicate that neuroinflammation in chronic migraine is focal and affects structures responsible for pain signaling (thalamus) and cognitive processing (hippocampus), which may contribute to central sensitization and cognitive impairment in patients.

## Figures and Tables

**Table 1:** Nucleotide sequences of primers for the assessment of gene expression levels by RT-PCR.

Gene, Full Name	Primer Name	Sequence	Product Lengh, nt
*Fos* (FBJ osteosarcoma oncogene)	Fos_Forward	5′-GAGCAGCTATCTCCTGAAGAGG-3′	130
	Fos_Reverse	5′-CAAGTTGATCTGTCTCCGCTTG-3′	
*Il1b* (interleukin 1 beta)	Il-1b_Forward	5′-CTTTGAAGAAGAGCCCGTCC-3′	221
	Il-1b_Reverse	5′-CGTTGCTTGTCTCTCCTTGTAC-3′	
*Il6* (interleukin 6)	Il-6_Forward	5′-CTCTGGTCTTCTGGAGTTCC-3′	179
	Il-6_Reverse	5′-GGAGAGCATTGGAAGTTGGG-3′	
*Nos2* (nitric oxide synthase 2, inducible)	Nos2_Forward	5′-CAGTGGCAACATCAGGTCG-3′	162
	Nos2_Reverse	5′-CACAACTGGGTGAACTCCAAG-3′	
*Tgfb1* (transforming growth factor, beta 1)	Tgf-b1_Forward	5′-GGCTTTCGCTTCAGTGCTC-3′	182
	Tgf-b1_Reverse	5′-GAGCACTGAAGCGAAAGCC-3′	
*Ifng* (interferon gamma)	Ifn-g_Forward	5′-CTGGCAAAAGGACGGTAACAC-3′	208
	Ifn-g_Reverse	5′-CTGTGGGTTGTTCACCTCG-3′	
*Vim* (vimentin)	Vim_Forward	5′-GTCCAAGTTTGCTGACCTCTC-3′	148
	Vim_Reverse	5′-CTCCAGGGACTCATTAGTGCC-3′	
*Il12a* (interleukin 12a)	Il-12a_Forward	5′-CACTCACATCTGCTGCTCC-3′	190
	Il-12a_Reverse	5′-ACTCAGGGGAACTGCTGCT-3′	
*Ptgs2* (prostaglandin-endoperoxide synthase 2)	Ptgs2_Forward	5′-GATCAGAAGCGAGGACCTGG-3′	175
	Ptgs2_Reverse	5′-CCTGAGTGTCTTTGACTGTGG-3′	
*P2rx7* (purinergic receptor P2X, ligand-gated ion channel, 7)	P2xr7_Forward	5′-GCACCATCAAGTGGATCTTGC-3′	231
	P2xr7_Reverse	5′-CAAAGAACGAGTTCCCCTGC-3′	
*Stat3* (signal transducer and activator of transcription 3)	Stat3_Forward	5′-GAAGAGTGCCTTCGTGGTG-3′	109
	Stat3_Reverse	5′-GACCAGCAACCTGACTTTTGTG-3′	
*Gapdh* (glyceraldehyde-3-phosphate dehydrogenase)	Gapdh_Forward	5′-CGGTGTGAACGGATTTGGC-3′	228
	Gapdh_Reverse	5′-GGATCTCGCTCCTGGAAGATG-3′	
*Actb* (actin, beta)	Actb_Forward	5′-GACCCAGATCATGTTTGAGACC-3′	104
	Actb_Reverse	5′-CCATCACAATGCCAGTGGTAC-3′	

## Data Availability

The data that support the findings of this study are available on request from the corresponding author.

## Supplementary Materials

The following supporting information can be downloaded at: https://www.mdpi.com/article/10.3390/ijms27010026/s1.

## Abbreviations

The following abbreviations are used in this manuscript:

AAV	Adeno-associated viruses
Actb	Actin, beta
BBB	Blood–brain barrier
CGRP	Calcitonin gene-related peptide
cpYFP	Circularly permuted yellow fluorescent protein
CSD	Cortical spreading depression
Gapdh	Glyceraldehyde-3-phosphate dehydrogenase
GFAP	Glial fibrillar acid protein
Hb	Hemoglobin
hSyn1	Human synapsin 1
Ifng	Interferon gamma
Il12a	Interleukin 12a
Il1b	Interleukin 1 beta
Il6	Interleukin 6
MC	Mast cells
MTS2	Mitochondrial targeting sequence 2
NO	Nitric oxide
Nos2	Nitric oxide synthase 2
NTG	Nitroglycerin
P2rx7	Purinergic receptor P2X, ligand-gated ion channel, 7
Ptgs2	Prostaglandin-endoperoxide synthase 2
ROS	Reactive oxygen species
Stat3	Signal transducer and activator of transcription 3
Tgf1b	Transforming growth factor beta 1
TNC	Trigeminal nucleus caudalis
Vim	Vimentin
