# Supplementary material for: Neuroinflammatory and Redox Responses in a Rat Model of NTG-Induced Migraine
Source: Int J Mol Sci. 2025 Dec 19;27(1):26. doi: 10.3390/ijms27010026 (PMC12785633; doi:10.3390/ijms27010026)
Supplement: Supplementary file 1 [file ijms-27-00026-s001.zip › ijms-3945850-supplementary/SUPPLEMENTARY MATHERIALS.pdf]

SUPPLEMENTARY MATHERIALS

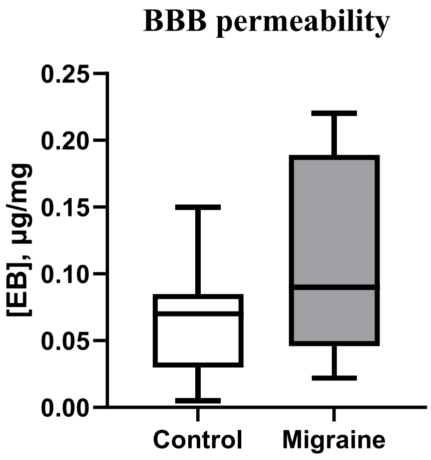

Supplementary Figure S1. Blood-brain barrier permeability in the chronic NTG-induced migraine rat model.

Supplementary Table S1. Comparative analysis of gene expression levels between control and NTG groups. Mann-Whitney U test, N=5 in group.

Brain tissue	Gene	Median (Control)	Median (NTG)	U, p=p-value
Striatum (STR)	*Fos*	0.2578	0.5777	7, p=0.3095
	Il1b	-0.1131	0.1040	11, p=0.8413
	Il6	0.1481	0.1874	10, p=0.6905
	Nos2	-0.5448	1.267	5, p=0.1508
	Tgfb1	-0.001580	0.4117	4, p=0.0952
	Ifng	0.5237	0.1668	12, p>0.9999
	Vim	0.2807	0.1529	11, p=0.8413
	Il12a	-0.4050	0.4345	12, p>0.9999
	Ptgs2	-0.4106	0.1682	10, p=0.6905
	P2rx7	0.3733	1.229	8, p=0.4206
	Stat3	0.2739	0.3393	7, p=0.3095
Hypothalamus (HIP)	*Fos*	-0.2527	0.1359	11, p=0.8413
	Il1b	-0.4958	-0.2766	12, p>0.9999
	Il6	0.1691	0.1081	12, p>0.9999
	Nos2	-0.3644	-0.4193	10, p=0.6905
	Tgfb1	-0.07184	0.09238	10, p=0.6905
	Ifng	0.05711	-0.5335	10, p=0.6905
	Vim	0.07875	-0.1650	10, p=0.6905
	Il12a	0.06852	-1.500	9, p=0.5476
	Ptgs2	0.2304	-0.2436	12, p>0.9999
	P2rx7	0.07356	-0.02303	12, p>0.9999
	Stat3	0.02490	0.1007	10, p=0.6905
Primary somatosensory cortex (S1)	*Fos*	-0.1170	0.8449	9, p=0.5476
	Il1b	-0.1686	0.4354	10, p=0.6905
	Il6	0.7704	0.4873	12, p>0.9999
	Nos2	-0.3604	-0.5288	12, p>0.9999
	Tgfb1	0.7157	1.108	6, p=0.2222
	Ifng	-0.5241	2.146	7, p=0.3095
	Vim	0.3087	0.5525	8, p=0.4206

	Il12a	-0.2105	1.851	6, p=0.2222
	Ptgs2	-0.07367	-0.2216	11, p=0.8413
	P2rx7	0.3262	0.4323	9, p=0.5476
	Stat3	0.1480	0.6352	7, p=0.3095
Trigeminal nuclei (TNC)	*Fos*	-0.08879	-0.1953	12, p>0.9999
	Il1b	0.2697	-0.9528	10, p=0.6905
	Il6	-0.1098	-1.093	7, p=0.3095
	Nos2	0.2528	-0.8075	10, p=0.6905
	Tgfb1	0.3610	-0.5767	9, p=0.5476
	Ifng	0.5745	-0.3368	12, p>0.9999
	Vim	0.2440	0.3740	9, p=0.5476
	Il12a	0.2198	0.2823	10, p=0.6905
	Ptgs2	1.137	1.409	4, p=0.0952
	P2rx7	-0.003550	-0.3531	7, p=0.3095
	Stat3	-0.01042	-0.1196	10, p=0.6905
Thalamus (TH)*	*Fos*	0.3289	-0.7796	4, p=0.0952
	Il1b	-0.2596	0.6077	8, p=0.4206
	Il6	0.08395	0.4009	9, p=0.5476
	Nos2	0.5789	2.362	8, p=0.4206
	Tgfb1	**0.09913**	**-0.4233**	**2, p=0.0317**
	Ifng	**0.9058**	**1.584**	**1, p=0.0159**
	Vim	-0.07449	0.3192	7, p=0.3095
	Il12a	-0.6477	0.7106	10, p=0.6905
	Ptgs2	0.2832	-1.065	10, p=0.6905
	P2rx7	-0.1954	-0.4549	10, p=0.6905
	Stat3	0.05649	-0.2039	11, p=0.8413

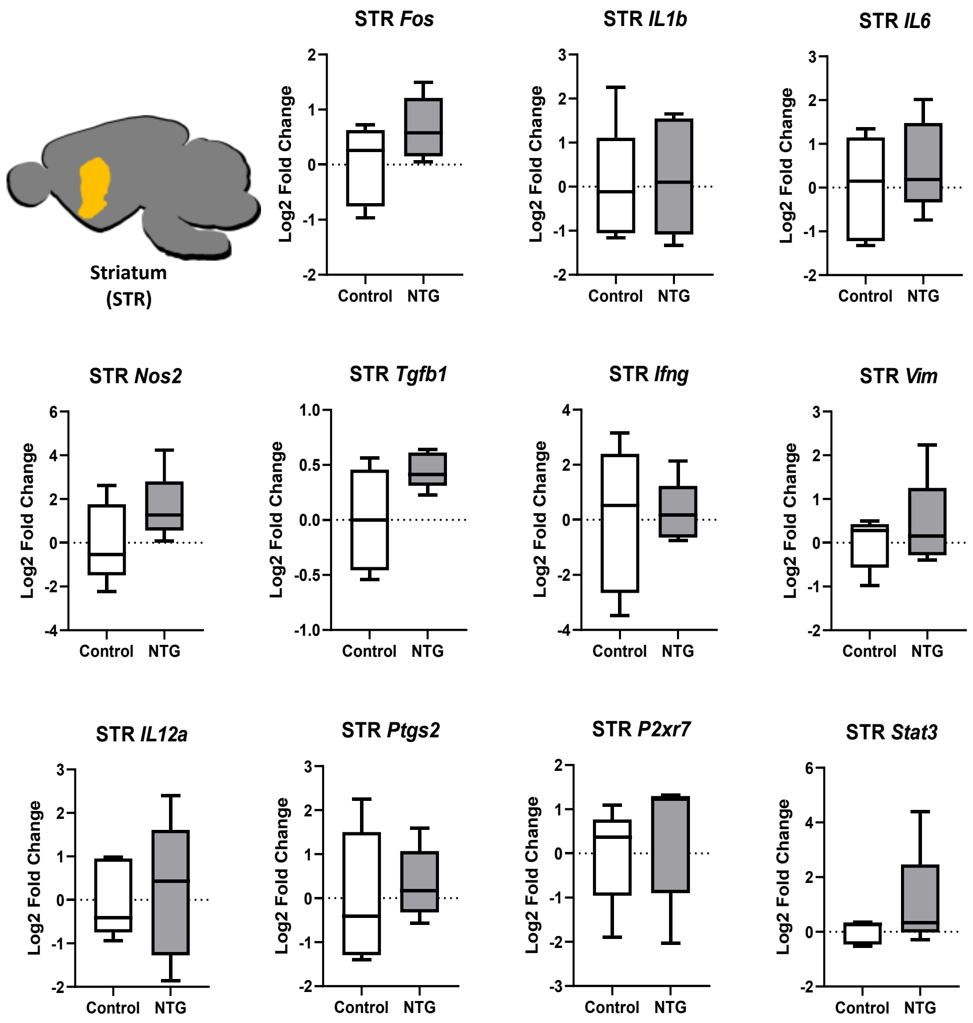

Supplementary Figure S2. Comparison of gene expression in the striatum in chronic migraine.

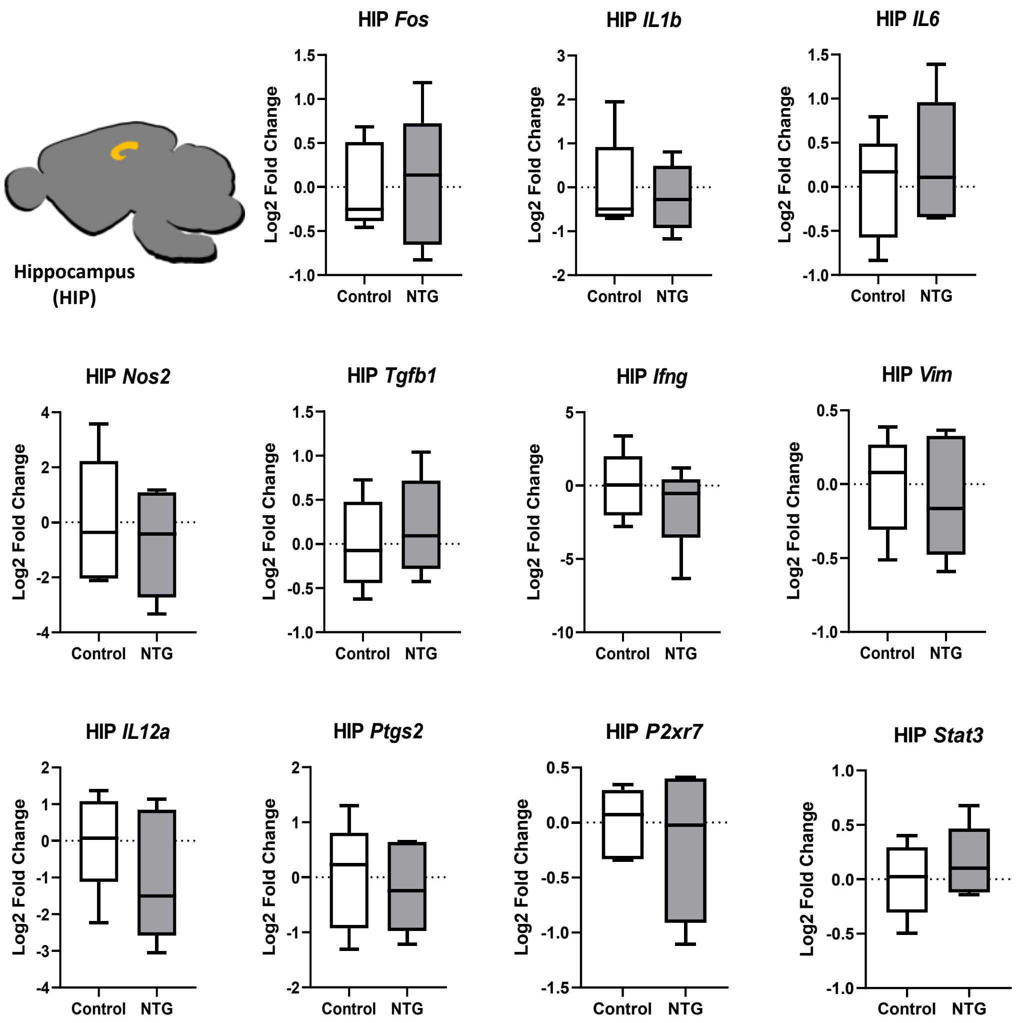

Supplementary Figure S3. Comparison of gene expression in the dorsal hippocampus in chronic migraine.

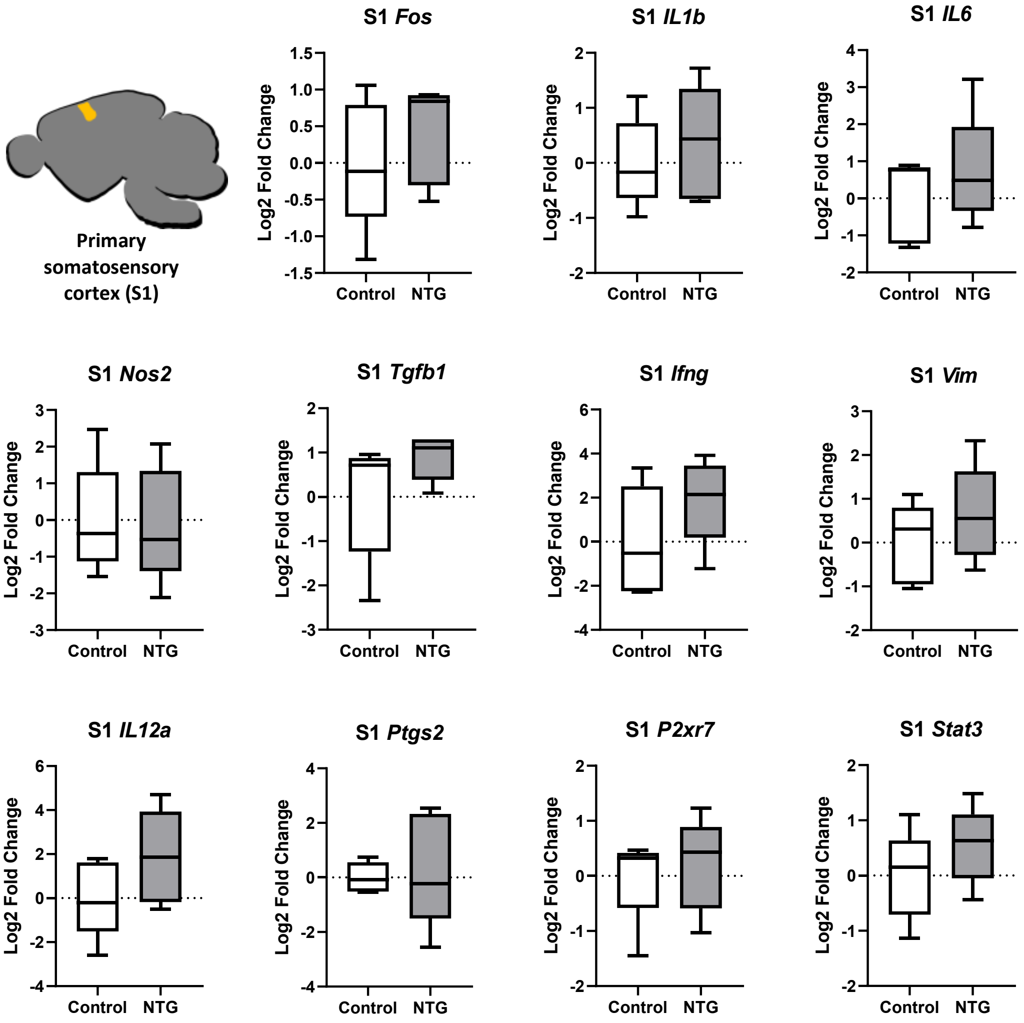

Supplementary Figure S4. Comparison of gene expression in the primary somatosensory cortex in chronic migraine.

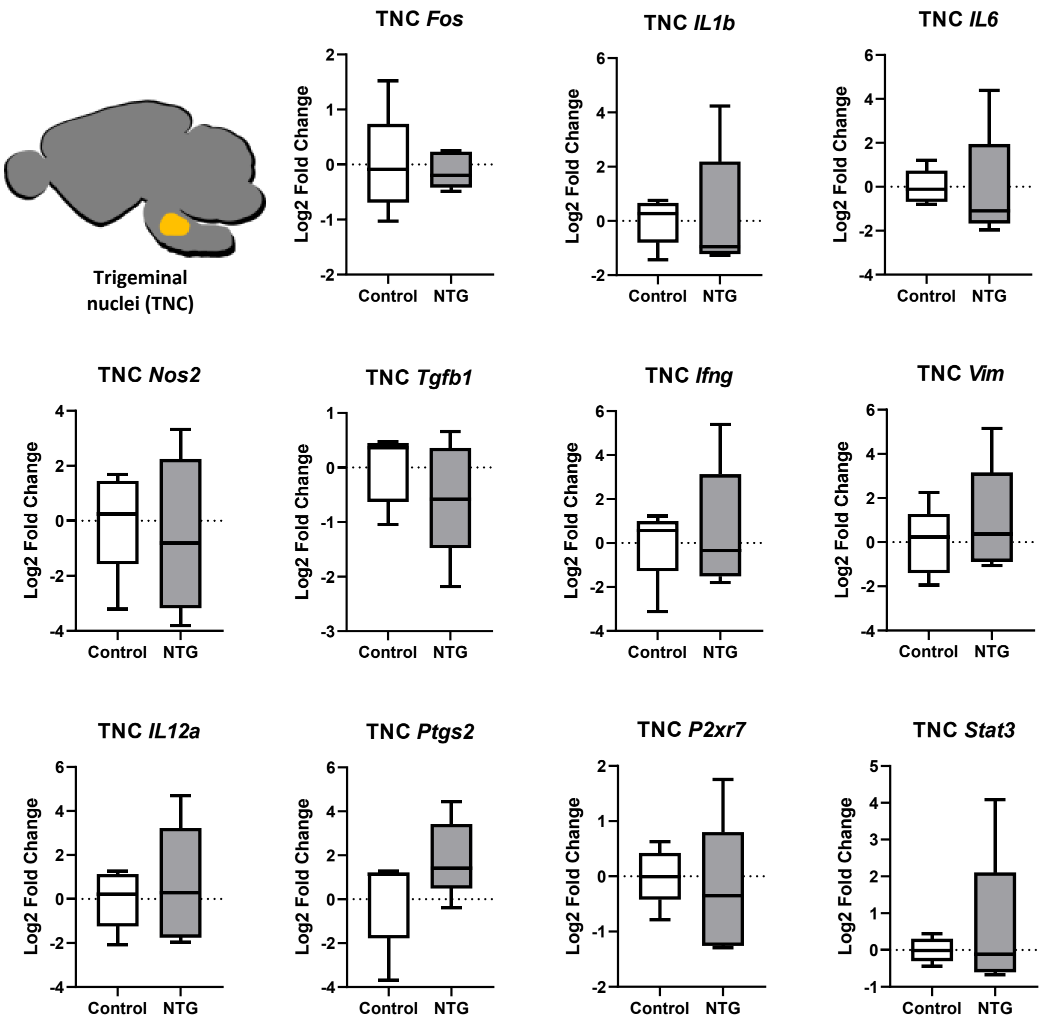

Supplementary Figure S5. Comparison of gene expression in the trigeminal nerve nuclei in chronic migraine.

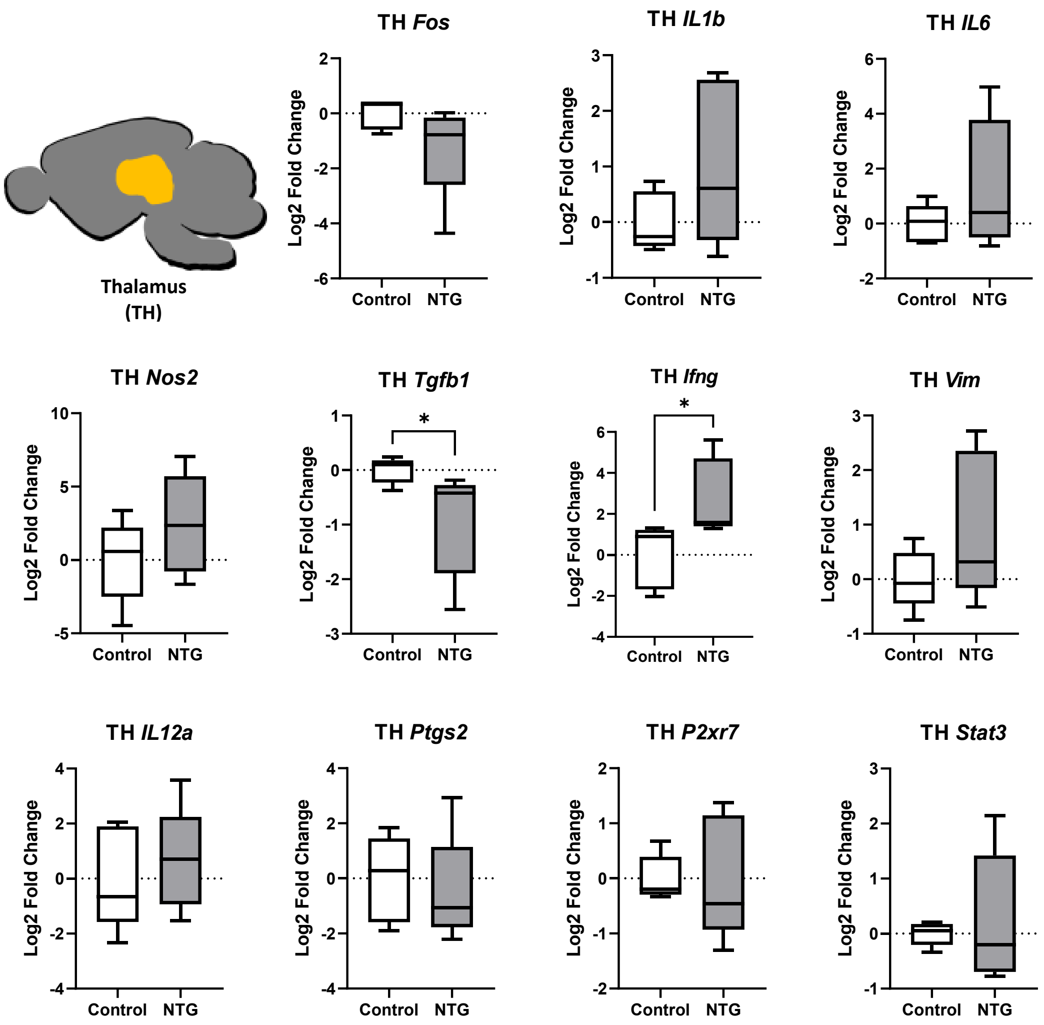

Supplementary Figure S6. Comparison of gene expression in the ventral thalamus in chronic migraine. * - $p < 0.05$, Mann–Whitney U test.
